# Supplementary material for: Revealing hole trapping in zinc oxide nanoparticles by time-resolved X-ray spectroscopy
Source: Nat Commun. 2018 Feb 2;9:478. doi: 10.1038/s41467-018-02870-4 (PMC5797134; doi:10.1038/s41467-018-02870-4)
Supplement: Supplementary file 1 — Supplementary Information [file 41467_2018_2870_MOESM1_ESM.pdf]

Supplementary Figures

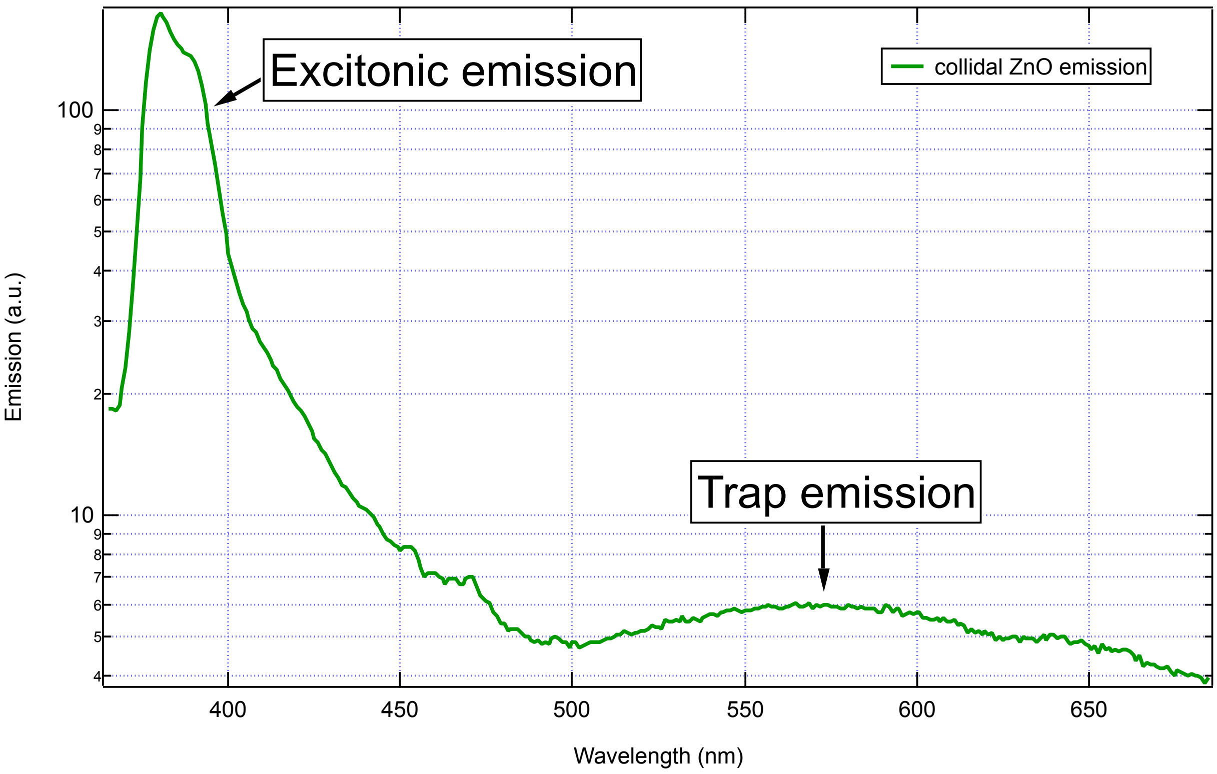

Supplementary Figure 1: UV and visible emission: Photoluminescence spectrum of the sample for excitation at 350 nm.

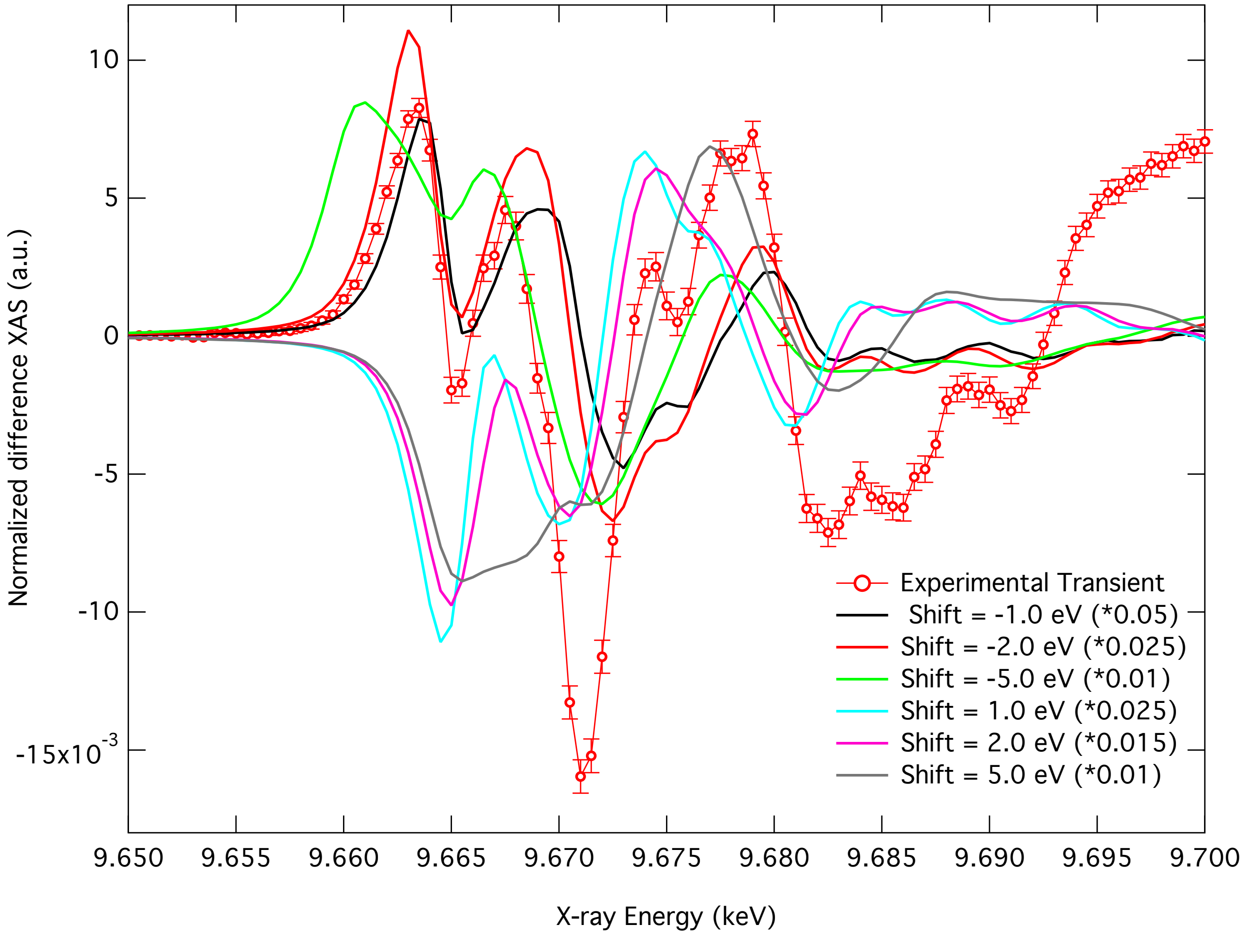

Supplementary Figure 2: Shifted Difference Spectrum: Comparison between the 80 ps Zn K-edge experimental transient spectrum and the shifted difference spectrum of [(ground state spectrum + shift eV) minus ground state spectrum]. Although for negative shifts, the simulated shifted difference spectrum captures the first two positive transient features, an edge shift leads to a derivative profile and therefore means that the simulation would also have to capture the negative feature. This is not the case, which rules out an edge-shift. Error bars shown are the standard error of the experimental measurement.

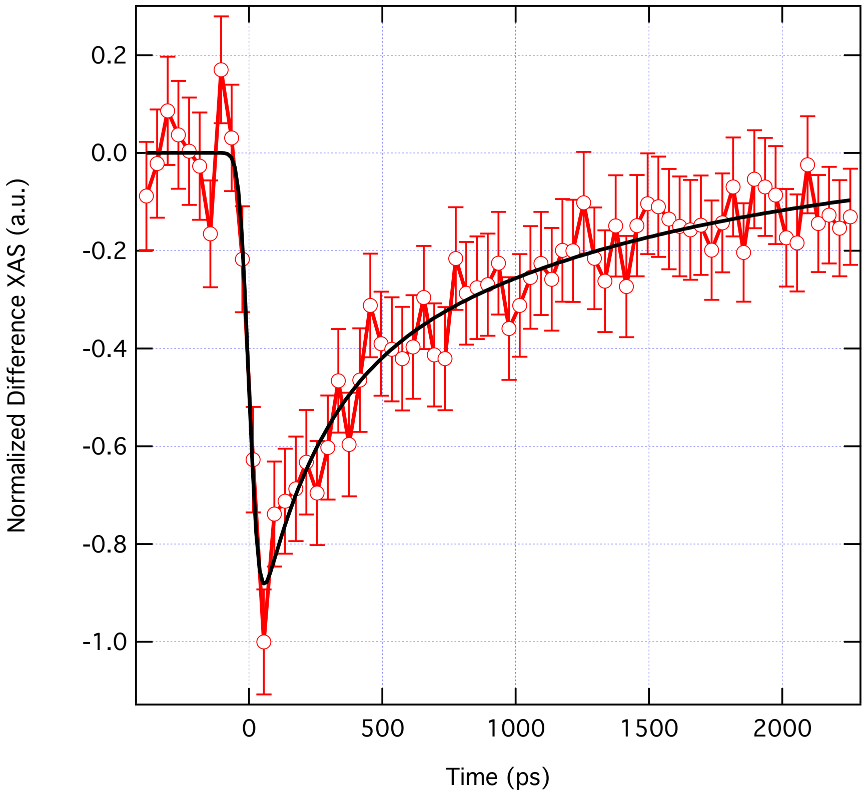

Supplementary Figure 3: Time scan: Kinetic trace with a biexponential fit (τ_1=200±128 ps and τ_2=1.2±0.3 ns) of the Zn K-edge transient XAS signal at 9.6703 keV. Error bars shown are the standard error of the experimental measurement.

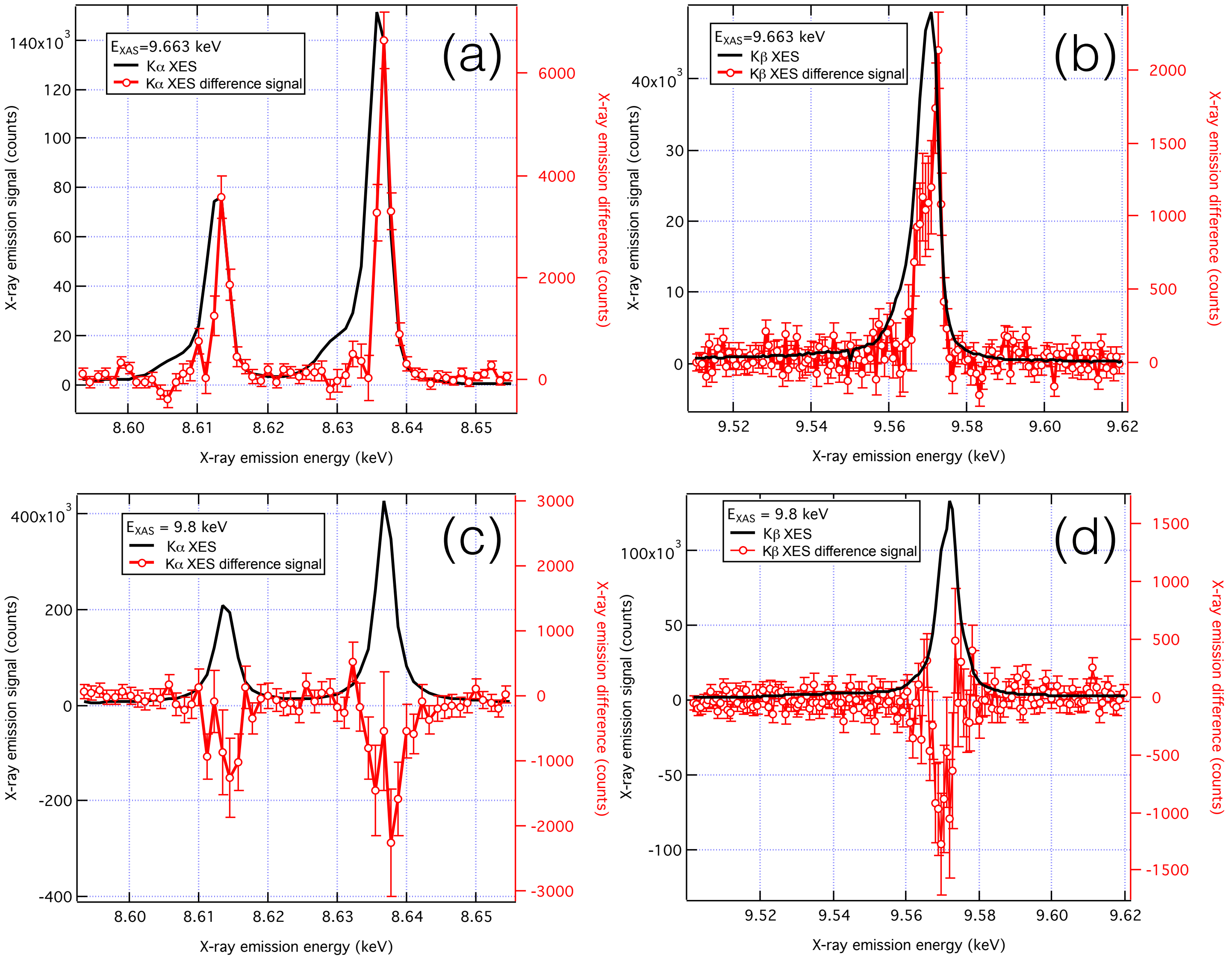

Supplementary Figure 4: Cuts of the Kα and Kβ RXES spectra: Ground state (red) and transient (black) Zn Kα RXES spectrum measured at incident X-ray energies of 9.663 keV (a) and 9.8 keV (c). Ground state (red) and transient (black) Zn Kβ RXES spectrum measured at incident X-ray energies of 9.663 keV (b) and 9.8 keV (d). Error bars shown are the standard error of the experimental measurement.

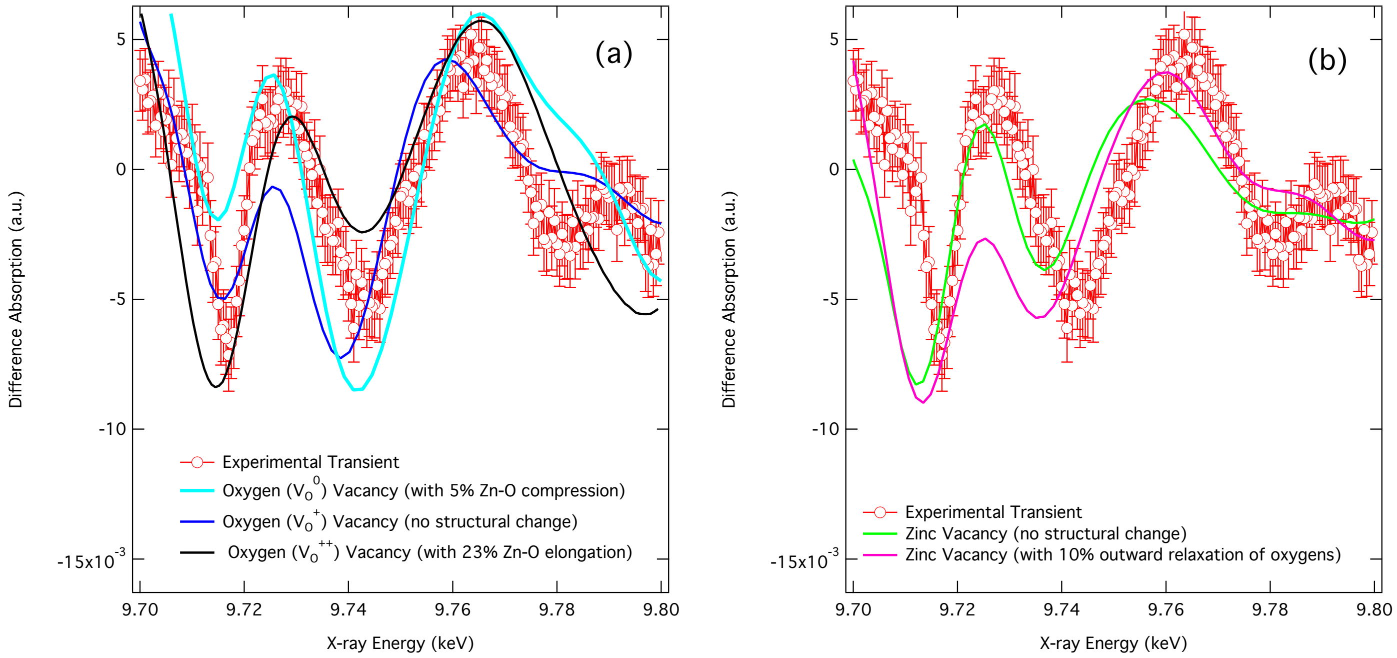

Supplementary Figure 5: Nature of the trapping site: Transient Zn K-edge EXAFS spectrum 80 ps after 355 nm photoexcitaton of 32 nm ZnO nanoparticles in colloidal solution compared to possible trapping site geometries for oxygen (a) and zinc (b) vacancies, with structures taken from Janotti et al. [7, 8] and using excitation levels of 7.5% and 2.5% respectively. The simulations were performed using the FEFF9 package, as described in the Supplementary Theory and Computation Details section. The root mean squared standard deviation (RMSD) of the calculated and experimental transients are: V_O^{2+} = 0.0017 (black), V_O^{+} = 0.0019 (blue), V_O^{0} = 0.0035 (cyan), V_{Zn} with outward distortion = 0.0027 (purple) and V_{Zn} no structural change = 0.0023 (green). Error bars shown are the standard error of the experimental measurement.

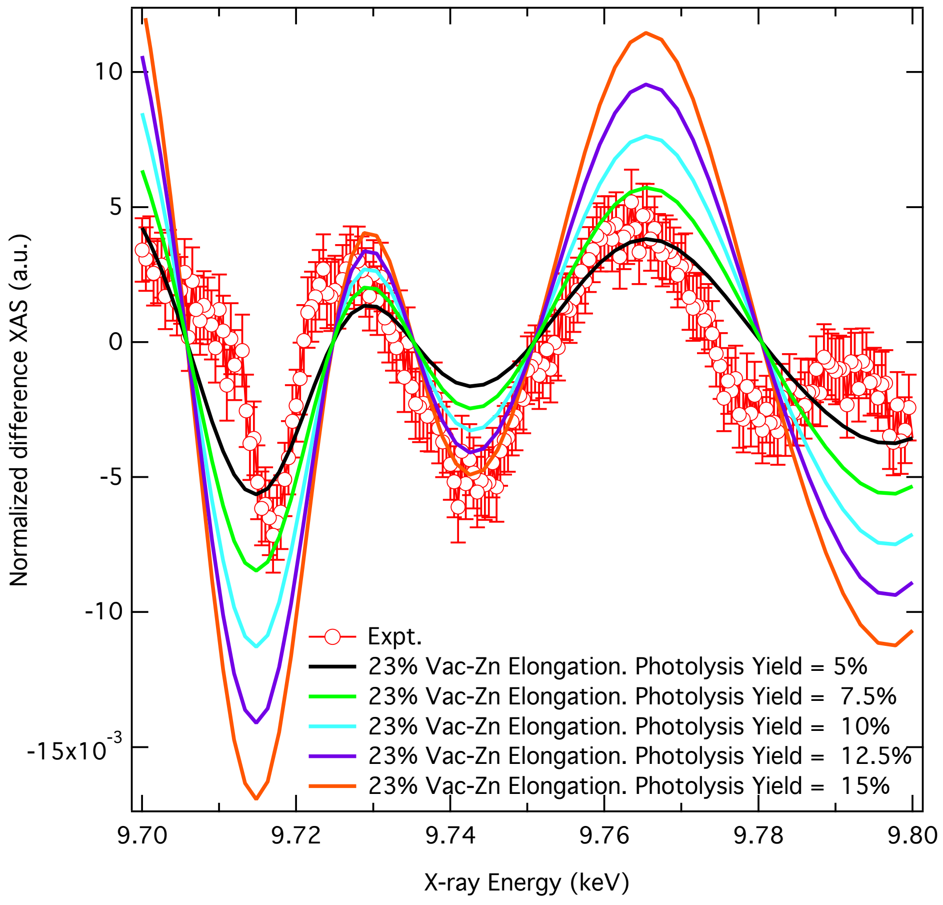

Supplementary Figure 6: Excitation fraction dependence of the V_O^{2+} defect: Transient Zn K-edge EXAFS spectrum 80 ps after 355 nm photoexcitaton of 32 nm ZnO nanoparticles in colloidal solution compared to the calculated transient for the V_O^{2+} defect using the DFT-calculated distortion of 23% [7] while varying the excitation fraction. The minimum RMSD of 0.0017 is found for f=7.5%. Error bars shown are the standard error of the experimental measurement.

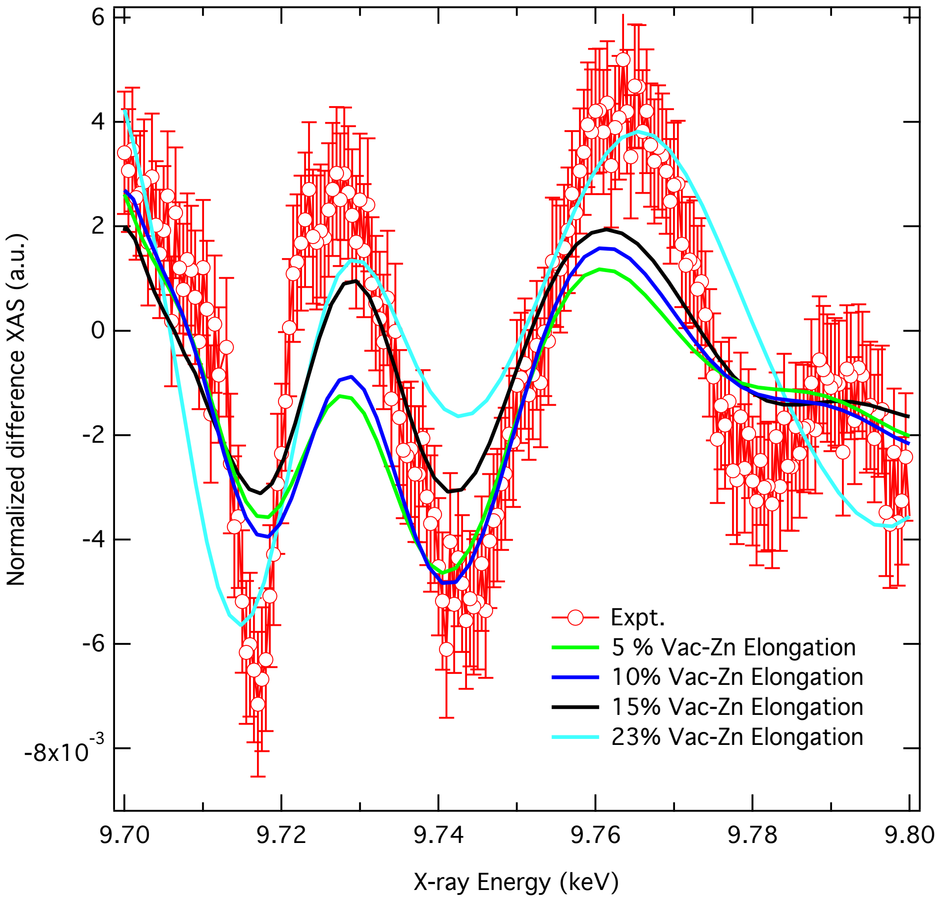

Supplementary Figure 7: Determining the Zn–Vacancy distortion: Transient Zn K-edge EXAFS spectrum 80 ps after 355 nm photoexcitaton of 32 nm ZnO nanoparticles in colloidal solution compared to the calculated transient for the V_O^{2+} defect with various Zn–Vacancy structural distortions and 7.5% excited state population optimised in Supplementary Figure 6. Error bars shown are the standard error of the experimental measurement.

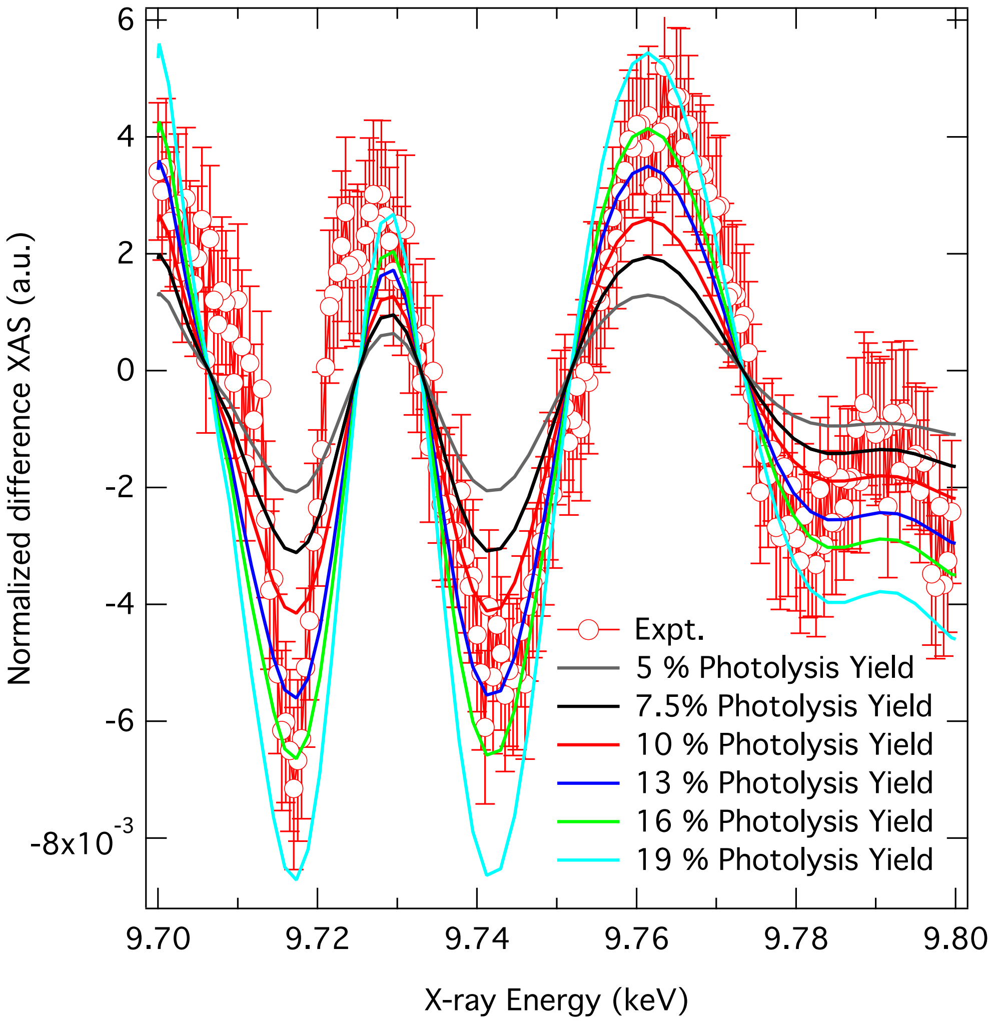

Supplementary Figure 8: Determining the Excited State Fraction: Transient Zn K-edge EXAFS spectrum 80 ps after 355 nm photoexcitaton of 32 nm ZnO nanoparticles in colloidal solution compared to the calculated transient for the V_O^{2+} defect at various excitation yields with a 15% structural distortion determined in Supplementary Figure 7. The 13% photolysis yield is the minimum RMSD for all calculations of 0.0012. Error bars shown are the standard error of the experimental measurement.

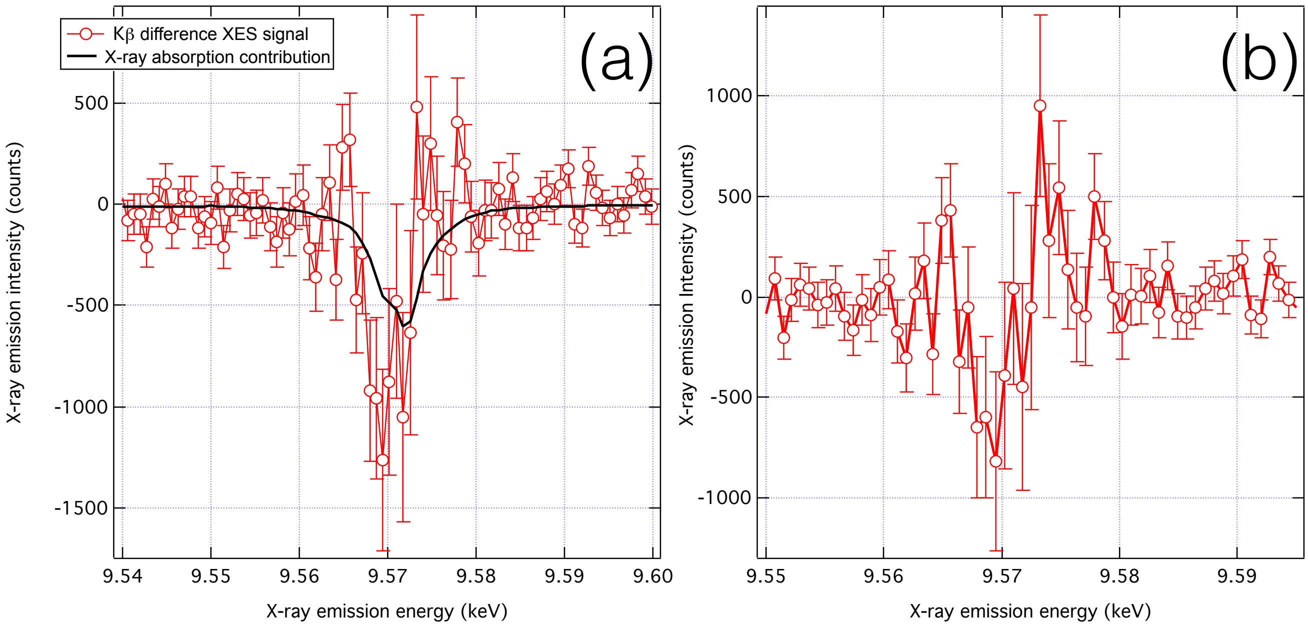

Supplementary Figure 9: Correcting the XES signals for XAS contribution: (a) The pump-probe difference XES signal for the Kβ emission signal (red) with the contribution from the change in XAS approximated to a change in amplitude of the Kβ emission (black). (b) The remaining XES difference signal after the XAS contribution has been removed showing a clear shift in X-ray emission energy. Error bars shown are the standard error of the experimental measurement.

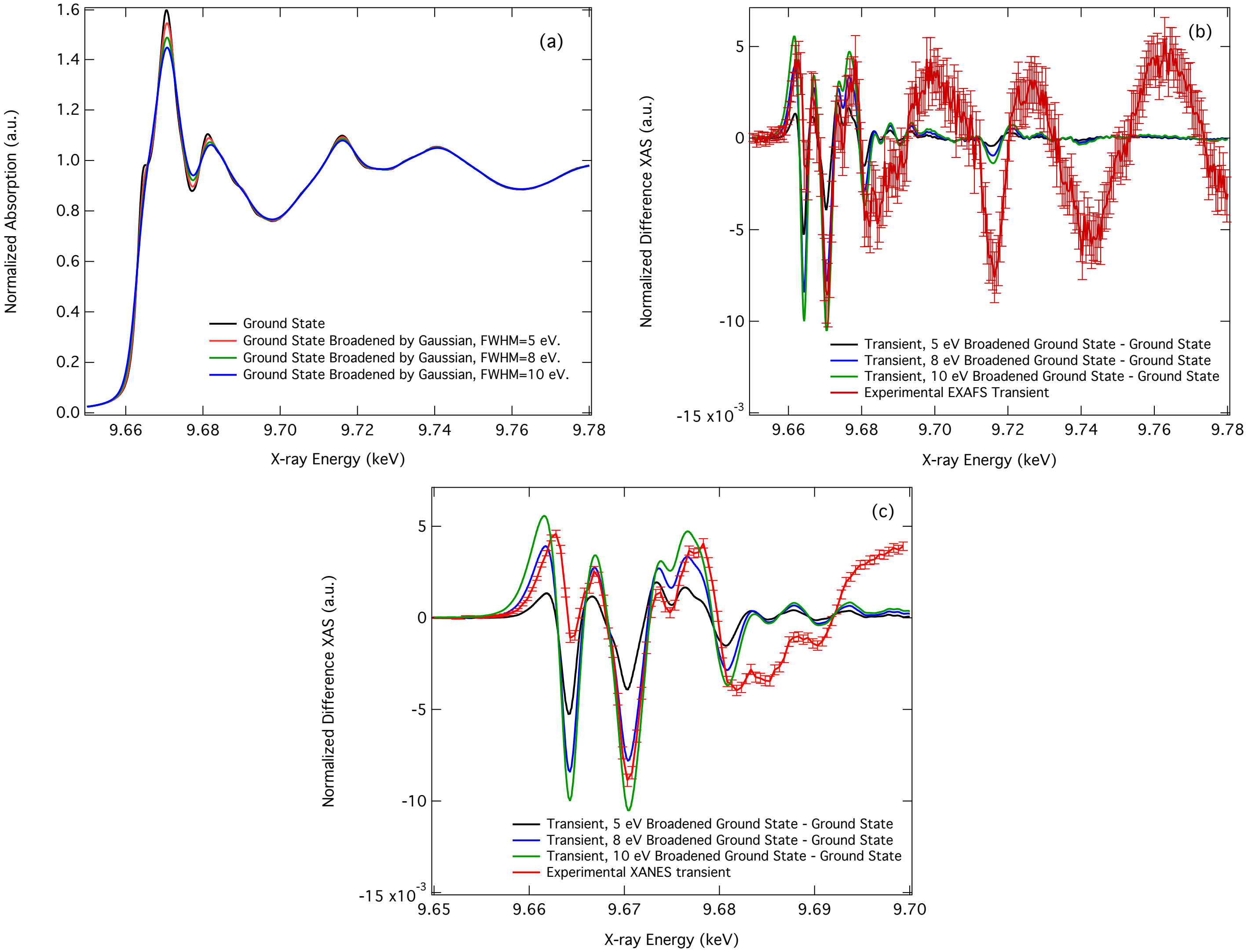

Supplementary Figure 10: Estimating the Transient: (a) Ground State Zn K-edge spectrum of 32 nm ZnO nanoparticles in aqueous solution shown with Gaussian-broadened ground state spectra (Full-Width-Half-Maximum=5, 8 or 10 eV) to simulate possible bleaching and disorder in the excited state. (b) Transient Zn K-edge EXAFS spectrum of 32 nm ZnO nanoparticles 80 ps after excitation at 355 nm (red trace) and the simulated spectra assuming that the excited state spectrum is purely a broadened ground state spectrum, as shown in (a). (c) The same simulated spectra shown in (b) in comparison to the XANES transient. Error bars shown are the standard error of the experimental measurement.

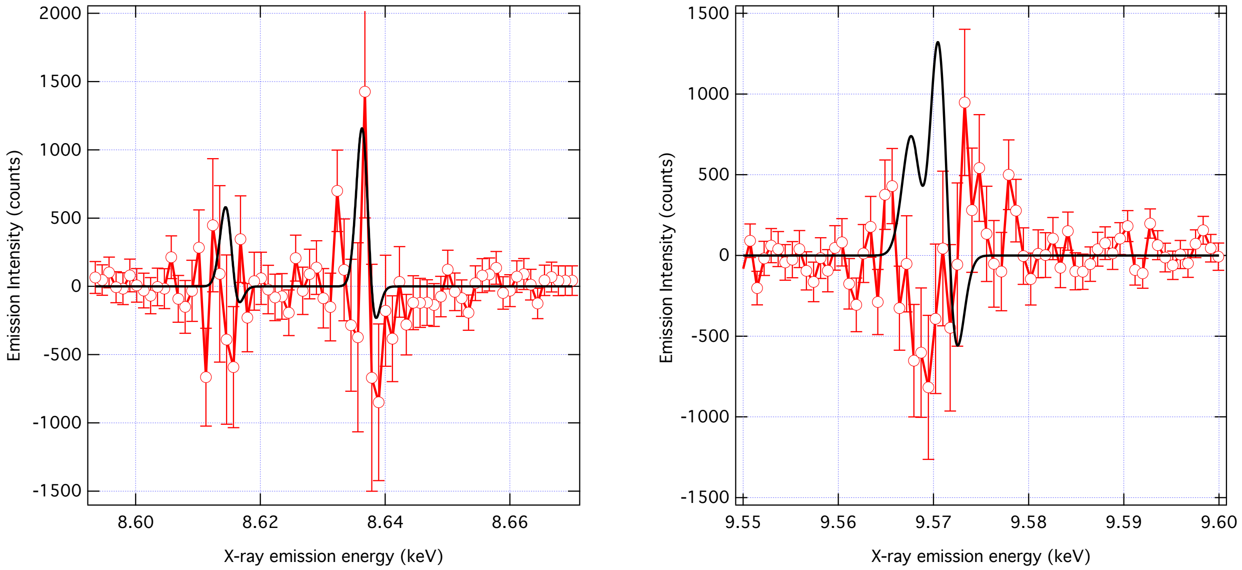

Supplementary Figure 11: Structural Sensitivity of XES spectra: The pump-probe difference XES signal for the Kα (left) and Kβ (right) emission signal (red) with the simulation for the V_{Zn} (black) with no structural distortion. Simulated difference signals shown correspond to an excitation of 2.5%. Error bars shown are the standard error of the experimental measurement.

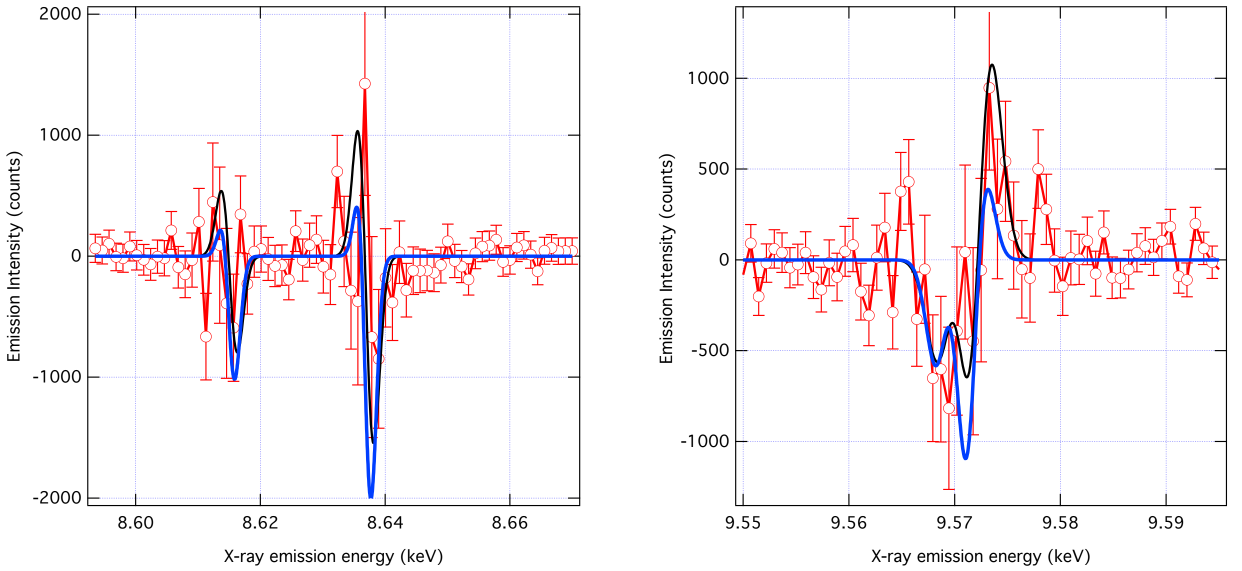

Supplementary Figure 12: Structural Sensitivity of XES spectra: The pump-probe difference XES signal for the Kα (left) and Kβ (right) emission signal (red) with the simulation for the V_O^{2+} (black) and the V_O^{+} (blue) vacancy. Simulated difference signals shown correspond to an excitation of 13%. Error bars shown are the standard error of the experimental measurement.

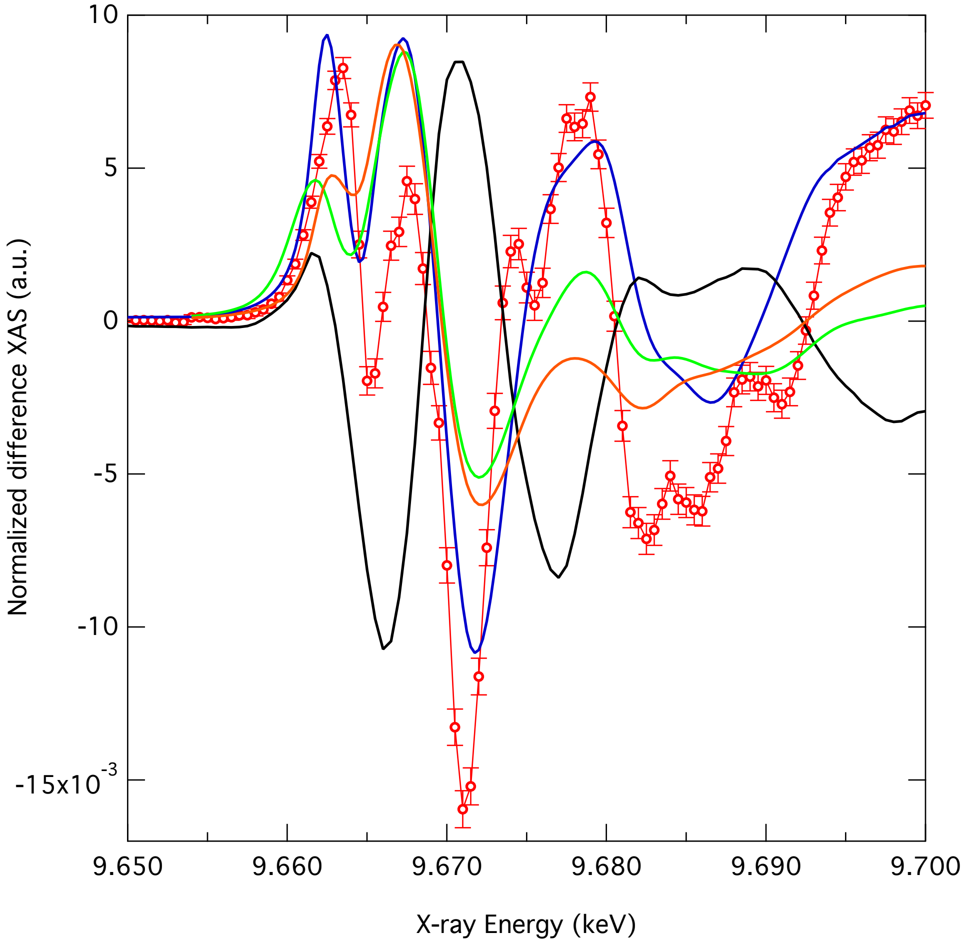

Supplementary Figure 13: Simulations of the Transient XANES Spectra: Transient Zn K-edge XANES spectrum 80 ps after 355 nm photoexcitaton of 32 nm ZnO nanoparticles in colloidal solution compared to the proposed trapping sites. The simulations were performed using the FDMNES package, as described in the Supplementary Theory and Computation Details section. Experimental spectrum is red, the V_O^{2+} with 15% distortion is blue, V_O^{+} is green, V_O^{0} is orange and V_{Zn} is black. Error bars shown are the standard error of the experimental measurement.

Supplementary Tables

	Band Gap (exitonic) luminescence						Green luminescence		
**Supplementary Reference**	**[26]**	**[27]**	**[28]**	**[29]**	**[30]**	**[31]**	**[32]**	**[33]**	**This work**
τ_1	20 ps	56 ps							
τ_2	200 ps	370 ps	400 ps		116 ps (428 ps)			347 ps	200 ps
τ_3				970 ps	585 ps (2.67 ns)	3.8 ns	2.47 ns	2.1 ns	1.2 ns
τ_4				14 ns			17.02 ns	18.8 ns	

Supplementary Table 1: List of literature emission lifetimes for the emission signals at various wavelengths for comparison to our results. Note that we have classified the lifetimes as a function of timescale: tens of ps (τ_1), hundreds of ps (τ_2), few ns (τ_3), and tens of ns (τ_4). Note: Numbers in brackets are after annealing.

Supplementary Methods

Supplementary Sample Information

The sample was a 170 mM dispersion of 32 nm diameter ZnO nanoparticles in water. It was prepared from a commercially available sample (Sigma-Aldrich 721077) which we have well characterized in a separate publication [1]. The UV-Vis emission from the sample when excited at 350 nm is shown in Supplementary Figure 1. We observed no changes in the sample over the duration of the experiment. Under these conditions the transmission of the jet was measured to be 33%, corresponding to an absorbance of 0.5 and an approximate excitation of 15% based simply on the number of species in the excitation volume.

Supplementary Experimental Setup Details

The experiment was performed at the 7ID-D beamline of the Advanced Photon Source, using their high-repetition rate laser and data acquisition setup [2]. The X-rays were focussed to a spot size of 7 μm (H) by 5 μm (V) using Kirkpatrick-Baez mirrors, and the laser spot size was 35 μm (all values FWHM). The sample was excited with 10 ps pulses at 355 nm (3.5 eV) at a fluence of 80 mJ/cm^2. The X-ray emission measurements were performed with a temporary installation of a two-crystal/two-detector setup to simultaneously measure the Kα and Kβ emission signals in a dispersive von Hamos geometry [3, 4] (See Figure 1 in the main text). The crystals were cylindrically bent Ge(220) and Si(111) with a 25 cm radius of curvature and the detectors were two Pilatus 100K modules. The total fluorescence yield was measured simultaneously with the XES using a scintillator and photomultiplier tube at normal incidence to the X-ray beam with an aperture to prevent detector saturation from the highly concentrated sample. The sample was recirculated through a 100 μm jet nozzle (Kyburz) to ensure a continuous flow of fresh sample using a pump. The sample was changed regularly to avoid problems with aggregation and any possible long-term sample changes.

Supplementary Theory and Computation Details

Simulations of the Zn K-edge absorption spectra

Calculations of the EXAFS region of the Zn K-edge spectrum were performed using multiple scattering and a self-consistent field (SCF) potential as implemented within FEFF9 [5]. The SCF loop is initialised by the construction of muffin-tin (MT) potentials where the MT-radii are chosen according to the Norman criterion [6]. A radius of 6.0 Å around the absorbing atom was used. For the excited state species, an oxygen atom was removed and the neighbouring zinc atoms moved as described in ref. [7]. No other atoms at larger distances from the defect were moved. This creates 4 inequivalent zinc atoms and therefore the excited state spectrum is a linear combination of the spectra from these four atoms. Once calculated, the transient (ΔA) is generated by subtraction of the ground state spectrum (A_{GS}) from the excited state (A_{ES}), which was scaled to match the experimental magnitude:

$$\Delta A = f(A_{ES} - A_{GS}) \tag{1}$$

This scaling (f) corresponds to the photolysis yield in the experiment and a value of $\sim$13±3% was found, as shown in Supplementary Figure 8. Besides the spectrum of the V_O^{2+} vacancy discussed in the main text, other proposed trapping sites [8] were tested and are shown in Supplementary Figure 5. It is noted that a double Zn vacancy [8] (i.e. two nearest neighbour Zn atoms missing) was also calculated. This gave an agreement with experiment which was only slightly worse than the V_O^{2+} vacancy, however in this case the photolysis yield would need to be about $\sim$75% to match the magnitude of the experimental transient. This is unrealistic considering the experimental parameters used herein.

Due to the limitations of the MT approximation close to the ionisation potential, calculations of the edge region of the spectrum were performed using the finite difference method as implemented within the Finite Difference Method Near Edge Scattering (FDMNES) package [9] using a free form SCF potential of radius 6.0 Å around

the absorbing atom. Broadening contributions due to the finite mean-free path of the photoelectron and to the core-hole lifetime were accounted for using an arctangent convolution [10]. Simulations of the transient spectrum were performed in the same manner as described in the previous paragraph. Due to the computational expense of these simulations, other trapping sites were not simulated. Instead, they were ruled out using the simulations of the EXAFS region shown in Supplementary Figure 5.

In general the excitation yield and the structural changes can be correlated in the transient XAS signal [11,12]. In this case the correlation was weak, allowing the shape of the transient XAS to be used to establish the Zn atom displacement away from the defect (15%, see Supplementary Figure 7) upon hole trapping, while the amplitude of the transient was used to estimate the excitation level ($\sim$13$\pm$3%, see Supplementary Figure 8). The XANES spectrum in particular is very sensitive to the magnitude of the displacement, and this was used for the fits. Janotti et al. used DFT to calculate the structural distortion due to the presence of the V_O^{2+} defect, resulting in a value of 23% for the Zn motion away from the vacancy [7]. Supplementary Figure 6 shows this structural distortion as a function of excitation yield, which clearly shows the lack of agreement for this structural distortion at all excitation levels.

Simulations of the Kα and Kβ X-ray emission

The X-ray emission spectra were simulated using the one-electron approach [13, 14] as implemented in the ORCA [15] quantum chemistry package. Computations used the BP86 functional [16, 17] and the def2-TZVP basis set [18, 19]. All of the calculations included spin orbit coupling (SOC), for which the SOC operator is approximated by the spin-orbit mean-field method (SOMF) [20]. After calculation a 2.5 eV broadening was applied to all calculated spectra to account for the intermediate and final state contributions.

The ZnO nanoparticle and associated defect site used for the calculation was described using a cluster model [21]. Here, the calculated system consisted of a $[Zn_{39}O_{38}]^{2+}$ cluster. This cluster was then embedded in an extended point charge field of just over 3000 atoms. For the point charge field, the formal charges for each atom were used, i.e. Zn=2+ and O=2-. To avoid erroneous electron delocalisation from the quantum region towards the point charge field region, the boundary between the two was described using repulsive capped effective core potentials.

Supplementary Note 1: The Transient RXES Cuts

Here we expand upon the discussion in the main text of the transient RXES spectra at incoming X-ray energies of 9.663 keV and 9.8 keV, respectively. Importantly, these spectra are represented using the Kramers-Heisenberg equation [22,23]:

$$F(\Omega,\omega)=\sum_f\left|\sum_n\frac{\langle f|\hat{H}_{int}|n\rangle\langle n|\hat{H}_{int}|i\rangle}{E_i-E_n-\hbar\Omega-i\dfrac{\Gamma_n}{2}}\right|^2\times\frac{\Gamma_f/2\pi}{(E_i-E_f+\hbar\Omega-\hbar\omega)^2+\Gamma_f^2/4} \tag{2}$$

where $\hbar\Omega$ and $\hbar\omega$ are the incident and emitted photons, respectively and E_i, E_n, E_f are the energies of the initial, intermediate and final states. Γ_n and Γ_f are the lifetime broadening associated with the intermediate and final states.

Eq. 2 describes the absorption of a photon, forming an intermediate state n from the ground state i, before it decays into a final state f. This second-order process can be described as absorption and emission processes that are coherently coupled. However, in the case of hard X-rays, these are generally small and the interference

terms can be neglected. Under this approximation Eq. 2 becomes:

$$F(\Omega,\omega) = \sum_f \sum_n \left| \frac{\langle f|\hat{H}_{int}|n\rangle\langle n|\hat{H}_{int}|i\rangle}{E_i - E_n + \hbar\Omega - i\frac{\Gamma_n}{2}} \right|^2 \times \frac{\Gamma_f/2\pi}{(E_i - E_f + \hbar\Omega - \hbar\omega)^2 + \Gamma_f^2/4} \tag{3}$$

$$= \sum_f \sum_n \frac{\langle f|\hat{H}_{int}|n\rangle^2\langle n|\hat{H}_{int}|i\rangle^2}{(E_i - E_n + \hbar\Omega)^2 + \frac{\Gamma_n^2}{4}} \times \frac{\Gamma_f/2\pi}{(E_i - E_f + \hbar\Omega - \hbar\omega)^2 + \Gamma_f^2/4} \tag{4}$$

This describes a non-coherent process in which the absorption matrix elements from initial state i to intermediate state n are weighted by the emission matrix element.

Consequently, the transient RXES spectra will reflect any changes in both the absorption ($\langle n|\hat{H}_{int}|i\rangle$) or emission matrix elements ($\langle f|\hat{H}_{int}|n\rangle$). As stated in the main text, by comparing with the transient TFY spectra, shown in Figure 2 in the main paper, we observe that the changes in these spectra reflect the changes in the absorption matrix elements. The dominance of the absorption ($\langle n|\hat{H}_{int}|i\rangle$) matrix elements in this case, reflects that the changes associated with the trapping of the photogenerated charge carrier are largely structural.

Supplementary Note 2: Analysis of XAS Results

The first step in the analysis was comparison of the experimental result to the so-called shifted difference spectrum, where the excited state is assumed to have a similar shape to the ground state XAS, but with a change in the position of the absorption edge, indicating a change in charge density around the absorbing atom. This technique can provide qualitative understanding of the photoexcited species [24, 25]. The result of this approach is shown in Supplementary Figure 2 where it is clear the agreement is poor, indicating a lack of change in charge density around the Zn atom in the excited state. The range of excitation levels used were from 1–5%, which were chosen to match the amplitude of the experimental transient. In order to rule out simple structural disorder in the excited state a similar approach was taken where the ground state spectrum was broadened, and then used as the simulated excited state. These results are shown in Supplementary Figure 10, where again the agreement is very poor with the experimental transient spectrum, indicating the photoexcited species is not simply a disordered ground state. The excitation level shown in Supplementary Figure 10 is 7%, which was chosen to match the amplitude of the experimental transient. In both experimentally-derived simulations of the excited state the agreement is sufficiently poor in the position of the transient features that no change in the photolysis yield would result in improved agreement (see Equation 1).

To identify the nature and the structure of the defect trapping site, we need to simulate the transient EXAFS, XANES and XES spectra. These simulated spectra depend sensitively on two parameters: the photolysis yield and the structural distortions (i.e. bond distances), which are obviously not known. Therefore, we proceeded by taking the DFT calculated structures [7, 8] for Zn and O vacancies in various charge trapping states, which consist of radial distortions (i.e. elongations or contractions) of the bond distances around the vacancies. These were then used for initial comparison to the experimental results starting with an approximate photolysis yield of 14% that was obtained from the absorbed laser fluence and sample concentration (see the Supplementary Experimental Setup Details and Supplementary Sample Information sections). After selecting the type of defect that comes closest to the EXAFS transient, we then explore the variation of the photolysis yield and determine the one that comes closest to reproducing the transient EXAFS. We then fix the photolysis yield to this value and vary the structural parameters of the defect to best fit the transient EXAFS. This iterative procedure between photolysis yield and bond distance variations allows us to narrow down the set of values that best reproduces the experimental transient EXAFS. These inputs are then used to simulate the transient XANES and XES spectra for consistency.

The results of EXAFS simulations of 5 different types of defects are shown in Figure 3 in the main paper and Supplementary Figure 5. In the latter, the agreement is clearly best for the V_O^{2+} defect as confirmed by

the root-mean-squared-deviation (RMSD) from the experimental transient. The dependence of the V_O^{2+} defect as a function of photolysis yield (Supplementary Figure 6) was then investigated, with the optimized photolysis yield than fixed while the Vacancy–Zn distortion distance was varied (Supplementary Figure 7). The photolysis yield was then re-investigated at the optimized Vacancy–Zn distortion distance (Supplementary Figure 8). These simulations confirmed the weak correlation between these two parameters, allowing them to be more easily disentangled. The result of this analysis indicated the best agreement with the EXAFS region of the spectrum was obtained with a Vacancy–Zn distortion of 15% and a photolysis yield of 13%.

To check this conclusion both the XANES region of the spectrum and the XES signals were simulated separately, using both the DFT-calculated defect structures and the optimized structure obtained from the EXAFS refinement. Further details on these simulations are provided in Supplementary Theory and Computation Details section. The results for the XANES region of the spectrum are shown in Supplementary Figure 13. The transients for V_O^+, V_O^0, and V_{Zn} are in poor agreement and can be ruled out, while the resulting simulation for the V_O^{2+} with 13% photolysis yield and 15% structural distortion provides the best agreement. The experimental measurement of the corrected XES difference signals (see Supplementary Note 3: The Correction of XES Difference Signals for the XAS Contribution for further details) contains large error bars, limiting its ability to be precisely compared to simulation. Three structures were simulated for comparison to experiment: the zinc defect V_{Zn}, the oxygen defect V_O^+, and the optimized V_O^{2+} structure. Even with the large experimental error bars it is clear the Zn vacancy disagrees with the experimental result (see Supplementary Figure 11), significantly for the Kβ XES, where the simulation shifts in the opposite direction from the experimental measurement. This is because the zinc vacancy, being about 3.5 Å from the absorbing atom, is too far to influence the inherently short range (3p,3d) exchange interaction. Supplementary Figure 12 shows the transient Kα and Kβ spectra for the V_O^{2+} and V_O^+ vacancies. The former is clearly in better agreement, although one should be careful of drawing too strong conclusions from this alone, due to the signal to noise ratio. However the agreement for the V_O^{2+} structure, when taken in combination with the XANES and EXAFS simulations, leads to our conclusion that the experimental results are all consistent with V_O^{2+} with 13% photolysis yield and 15% structural distortion.

Supplementary Note 3: The Correction of XES Difference Signals for the XAS Contribution

The primary approximation made here is that the total integrated counts in the X-ray emission peaks should change in the pump-probe signal by the same ratio measured in the total fluorescence yield difference signal. By evaluating the change in total XES counts at 9.8 keV due to laser excitation we obtain a difference ratio in the total counts which we can then apply to correct the X-ray emission signals shown in Supplementary Figure 4 for the change in total X-ray absorption cross-section. By removing this contribution, simply by scaling the emission peaks by this amount and removing this difference from the pump-probe XES signal, we can see any remaining laser-induced changes in the emission signals. The XAS signal contribution and its removal are shown in Supplementary Figure 9 for the Kβ X-ray emission difference signal.

Supplementary References

[1] Rossi, T. *et al.* Characterizing the Structure and Defect Concentration of ZnO Nanoparticles in a Colloidal Solution. *The Journal of Physical Chemistry C* **118**, 19422–19430 (2014).

[2] March, A. M. *et al.* Development of high-repetition-rate laser pump/x-ray probe methodologies for synchrotron facilities. *Review Of Scientific Instruments* **82**, 073110 (2011).

[3] Szlachetko, J. *et al.* A von Hamos x-ray spectrometer based on a segmented-type diffraction crystal for single-shot x-ray emission spectroscopy and time-resolved resonant inelastic x-ray scattering studies. *Review Of Scientific Instruments* **83**, 103105 (2012).

[4] Szlachetko, J. *et al.* Communication: The electronic structure of matter probed with a single femtosecond hard x-ray pulse. *Structural Dynamics* **1**, 021101 (2014).

[5] Rehr, J. *et al.* Ab initio theory and calculations of X-ray spectra. *Comptes Rendus Physique* **10**, 548 (2009).

[6] Norman, J. G. Non-empirical versus empirical choices for overlapping-sphere radii ratios in SCF-Xα-SW calculations on ClO_4 - and SO_2. *Molecular Physics* **31**, 1191–1198 (1976).

[7] Janotti, A. & Van de Walle, C. G. Oxygen vacancies in ZnO. *Applied Physics Letters* **87**, 122102 (2005).

[8] Janotti, A. & Van de Walle, C. G. Native point defects in ZnO. *Physical Review B* **76**, 165202 (2007).

[9] Joly, Y. X-ray absorption near-edge structure calculations beyond the muffin-tin approximation. *Physical Review B* **63**, 125120 (2001).

[10] Bunău, O. & Joly, Y. Self-consistent aspects of x-ray absorption calculations. *Journal of Physics-Condensed Matter* **21**, 345501 (2009).

[11] van der Veen, R. *et al.* Structural determination of a photochemically active diplatinum molecule by time-resolved EXAFS spectroscopy. *Angewandte Chemie* **48**, 2711–2714 (2009).

[12] Gawelda, W. *et al.* Structural analysis of ultrafast extended x-ray absorption fine structure with subpicometer spatial resolution: Application to spin crossover complexes. *The Journal of Chemical Physics* **130**, 124520 (2009).

[13] Beckwith, M. A. *et al.* Manganese Kβ X-ray Emission Spectroscopy As a Probe of Metal–Ligand Interactions. *Inorganic chemistry* **50**, 8397–8409 (2011).

[14] Lee, N., Petrenko, T., Bergmann, U., Neese, F. & DeBeer, S. Probing valence orbital composition with iron Kβ X-ray emission spectroscopy. *Journal of the American Chemical Society* **132**, 9715–9727 (2010).

[15] Neese, F. The ORCA program system. *Wiley Interdisciplinary Reviews-Computational Molecular Science* **2**, 73–78 (2012).

[16] Becke, A. Density-functional exchange-energy approximation with correct asymptotic behavior. *Phys. Rev. A* **38**, 3098–3100 (1988).

[17] Perdew, J. P. Density-Functional Approximation for the Correlation-Energy of the Inhomogeneous Electron-Gas. *Physical Review B* **33**, 8822–8824 (1986).

[18] Schäfer, A., Horn, H. & Ahlrichs, R. Fully optimized contracted Gaussian basis sets for atoms Li to Kr. *The Journal of Chemical Physics* **97**, 2571–2577 (1992).

[19] Weigend, F. & Ahlrichs, R. Balanced basis sets of split valence, triple zeta valence and quadruple zeta valence quality for H to Rn: design and assessment of accuracy. *Physical Chemistry Chemical Physics* **7**, 3297–3305 (2005).

[20] Hess, B. A., Marian, C. M., Wahlgren, U. & Gropen, O. A mean-field spin-orbit method applicable to correlated wavefunctions. *Chemical Physics Letters* **251**, 365–371 (1996).

[21] Maganas, D. *et al.* First principles calculations of the structure and V L-edge X-ray absorption spectra of V_2O_5 using local pair natural orbital coupled cluster theory and spin–orbit coupled configuration interaction approaches. *Physical Chemistry Chemical Physics* **15**, 7260–7276 (2013).

[22] Kramers, H. & Heisenberg, W. Über die Streuung von Strahlung durch Atome. *Z. Phys.* **31**, 681 (1925).

[23] Ament, L. J. P., van Veenendaal, M., Devereaux, T. P., Hill, J. P. & van den Brink, J. Resonant inelastic x-ray scattering studies of elementary excitations. *Reviews Of Modern Physics* **83**, 705–767 (2011).

[24] Rittmann-Frank, M. H. *et al.* Mapping of the Photoinduced Electron Traps in TiO_2 by Picosecond X-ray Absorption Spectroscopy. *Angewandte Chemie International Edition* **53**, 5858–5862 (2014).

[25] Santomauro, F. G. *et al.* Femtosecond X-ray absorption study of electron localization in photoexcited anatase TiO_2. *Scientific Reports* **5**, 14834 (2015).

[26] Danhara, Y., Hirai, T., Harada, Y. & Ohno, N. Exciton luminescence of ZnO fine particles. *physica status solidi c: Current Topics in Solid State Physics* **3**, 3565–3568 (2006).

[27] Xiong, G., Pal, U. & Serrano, J. G. Correlations among size, defects, and photoluminescence in ZnO nanoparticles. *Journal Of Applied Physics* **101**, 024317 (2007).

[28] Wilkinson, J., Ucer, K. B. & Williams, R. T. Picosecond excitonic luminescence in ZnO and other wide-gap semiconductors. *Radiation Measurements* **38**, 501–505 (2004).

[29] Koida, T. *et al.* Correlation between the photoluminescence lifetime and defect density in bulk and epitaxial zno. *Applied Physics Letters* **82**, 532–534 (2003).

[30] Teke, A. *et al.* Excitonic fine structure and recombination dynamics in single-crystalline zno. *Physical Review B* **70**, 195207 (2004).

[31] Chichibu, S. *et al.* Improvements in quantum efficiency of excitonic emissions in zno epilayers by the elimination of point defects. *Journal of applied physics* **99**, 93505–93505 (2006).

[32] Kamat, P. V. & Patrick, B. Photophysics and Photochemistry of Quantized ZnO Colloids. *The Journal of Physical Chemistry* **96**, 6829–6834 (1992).

[33] Bauer, C., Boschloo, G., Mukhtar, E. & Hagfeldt, A. Ultrafast relaxation dynamics of charge carriers relaxation in ZnO nanocrystalline thin films. *Chemical Physics Letters* **387**, 176–181 (2004).
